# An accurate semantic segmentation model for bean seedlings and weeds identification based on improved ERFnet

**DOI:** 10.1038/s41598-024-61981-9

**Published:** 2024-05-29

**Authors:** Haozhang Gao, Mingyang Qi, Baoxia Du, Shuang Yang, Han Li, Tete Wang, Wenyu Zhong, You Tang

**Affiliations:** 1 Electrical and Information Engineering College, Jilin Agricultural Science and Technology University, Jilin, 132101 China; 2 School of Information and Control Engineering, Jilin Institute of Chemical Technology, Jilin, 132022 China; 3 R &D Department, Jilin Province Electric Innovation Information Technology Limited Company, Changchun, 130117 China

**Keywords:** Computer science, Information technology

## Abstract

In agricultural production activities, the growth of crops always accompanies the competition of weeds for nutrients and sunlight. In order to mitigate the adverse effects of weeds on yield, we apply semantic segmentation techniques to differentiate between seedlings and weeds, leading to precision weeding. The proposed EPAnet employs a loss function coupled with Cross-entropy loss and Dice loss to enhance attention to feature information. A multi-Decoder cooperative module based on ERFnet is designed to enhance information transfer during feature mapping. The SimAM is introduced to enhance position recognition. DO-CONV is used to replace the traditional convolution Feature Pyramid Networks (FPN) connection layer to integrate feature information, improving the model’s performance on leaf edge processing, and is named FDPN. Moreover, the Overall Accuracy has been improved by 0.65%, the mean Intersection over Union (mIoU) by 1.91%, and the Frequency-Weighted Intersection over Union (FWIoU) by 1.19%. Compared to other advanced methods, EPAnet demonstrates superior image segmentation results in complex natural environments with uneven lighting, leaf interference, and shadows.

## Introduction

Green bean, scientifically known as Phaseolus vulgaris L., holds significant importance as a vegetable crop in numerous developing nations, serving as a valuable protein and nutrient source. According to the Food and Agriculture Organization (FAO) report^1^, the global cultivation area dedicated to green beans amounts to 1,527,613 hectares, resulting in an impressive production of 21,720,588 tons. China occupies the leading position as the world’s primary producer of green beans, with a substantial cultivated area spanning 635,385 hectares. Notably, China’s green bean production stands at an astounding 17,031,702 tons^1^. This underscores China’s pivotal position as the foremost producer of green beans globally, emphasizing the widespread impact of this vegetable crop in meeting nutritional needs and supporting food security, particularly in developing countries. However, the final yield of beans is influenced by various growth factors and climate conditions^2^, with the negative effects of weeds being particularly significant. And weeds not only compete for the soil fertility required during the growth of bean seedlings but also promote diseases that jeopardize the seedlings^3^. Therefore, the agricultural sector urgently needs to accurately distinguish between crops and weeds to effectively address the weed problem. The conventional weeding techniques predominantly depend on pesticide application, which, besides its restricted efficacy, can result in the wastage of manpower and environmental contamination^4^. As computer technology perpetually evolves, deep learning and computer vision methodologies have garnered extensive utilization. In pursuit of pollution-free weeding, researchers are required to pinpoint the best image segmentation techniques for distinguishing crops from weeds, enhance identification precision, assist robots in accurate weeding. Semantic segmentation of images remains a critical aspect of computer vision endeavors. This direction also stands as a key research topic for numerous computer scholars globally. The application scenarios for semantic segmentation are vast, including autonomous driving of vehicles^5–7^, identification and recognition of medical images^8^, urban planning^3^, and smart agriculture^9^, among others. By removing the fully connected modules, semantic segmentation successfully realizes the pixel-by-pixel of the fully convolutional networks. However, the performance of fully convolutional networks during the inference phase is not ideal. In subsequent work, by addressing the intrinsic association issues of semantic positions, the inference results were noticeably improved. Semantic segmentation models commonly adopt an encoder-decoder structure. The encoder primarily focuses on learning and refining image features, while the decoder performs pixel-level categorization of these features. To capture the semantics in images more deeply, the encoder typically consists of several convolutional layers. For instance, UNet is a classic encoder-decoder structured algorithm that employs a skip-connection strategy, effectively achieving feature fusion and thereby addressing potential information loss during the downsampling process^10^. The downsampling of features plays a pivotal role in the model^11^. However, considering computational costs, the resolution of feature maps is often reduced, leading to more abstract features extracted by the encoder. And the receptive field of the model also significantly expands^12^. The attention mechanism module is frequently integrated with the Fully Convolutional Networks (FCN) framework in the architecture of semantic segmentation models^13,14^, and this has led to the derivation of various multi-level feature fusion model variants^15,16^. For example, Deeplabv3, as one of the Deeplab series models, has a more streamlined and efficient decoder module, which allows for more detailed feature extraction and simultaneously enhances semantic segmentation performance^11,17^. It introduces dilated convolution to expand the receptive field, while PSPNet employs parallel pooling at different scales to extract various types of features, thereby enhancing the model’s segmentation results^18^.

Deep learning extensive application in image segmentation has become evident^19^. The pace of deep learning has accelerated, starting from fully convolutional networks, but there’s still substantial room for improvement in segmentation algorithms^9^. Deep learning algorithms based on convolutional neural networks made significant progress in the field of plant phenomics, especially in the application of plant leaf segmentation^20^. Mehdipour et al. proposed a simplified CNN model in the 2015 plant species identification competition^21^. The core idea of this model is to learn weights through principal component analysis with mean removal, achieving over 90% plant leaf recognition rate in images. However, this method slightly falls short in recognizing leaf images with complex backgrounds. Daniel D. Morris proposed a multi-scale pyramid convolutional neural network for segmenting densely packed plant leaves, allowing for more precise delineation of leaf areas in images^22^. Dmitry Kuznichov et al. introduced a method focused on preserving the geometric structural information of plant leaves to approximate real scenarios^23^. And they employed non-maximum suppression to address the occlusion problem during the training of FCN, achieving precise leaf segmentation in dense scenarios. Although the studies mentioned have shown advancements in the realm of plant leaf segmentation, optimal results remain elusive.

Under natural conditions, influenced by factors like natural lighting, weather conditions, the angle of the image sensor, and interference from surrounding leaves^24^, how to effectively perform image segmentation under such conditions^25^has become an urgent problem to address. To confront this challenge in the context of natural conditions, Zheng L and his team used algorithms like Mean-shift and BP neural network to segment leaves from different vegetables, yielding impressive outcomes^26^. To improve the accuracy of plant leaf segmentation, many people have conducted studies based on features like the shape and color of the leaves. Omrani et al. utilized the K-means clustering algorithm to identify and segment various plant leaves^27^, and employed the features of cluster centers for precise segmentation and perfectly mapped the results to the original images. Additionally, they also employed support vector machines to classify the leaves. In 2020, Praveen Kumar et al. proposed an efficient leaf segmentation algorithm based on Deep Convolutional Neural Networks (DCNN), and extracted leaf features by DCNN and applied orthogonal transformation techniques for precise segmentation of specific plant leaves^28^. Notably, during the segmentation process, they also utilized the CMYK color space for denoising, further enhancing the efficiency of edge detection for leaves^29^.

In summary, beans, as significant agricultural crops, play a crucial role in human society. However, weeds seriously impact their growth and yield. The role of image segmentation in environmentally-friendly weed control becomes increasingly significant. People continuously explore various algorithms and methods to achieve precise image segmentation between crops and weeds, providing valuable support for the future development of agriculture. In this context, we propose the EPAnet algorithm for semantic segmentation of crops and weeds. Moreover, the algorithm presented in this paper has improved the Overall Accuracy by 0.65% compared to the benchmark model ERFnet, with a 1.91% increase in mIoU and a 1.19% increase in FWIoU. The contributions of this paper are:

1. Expanding public datasets can enhance generalization capabilities and accuracy of the model.
2. Adopting a dual-loss coupled function as the loss function stabilizes the gradient descent process and accelerates convergence while balancing class imbalance.
3. The SimAM is incorporated during the downsampling phase, helping the model focus on the parts of the image most relevant to the task, thus avoiding interference from irrelevant information.
4. Given the design of ERFnet as a real-time segmentation algorithm, it intentionally omits some information during the downsampling to increase its operational speed, which is a primary reason for its less-than-ideal accuracy. In consideration of precision, we propose a new FDPN connection module to enhance the information exchange during both the downsampling and upsampling stages, thus elevating the accuracy of the algorithm.
5. In the decoder section, fusion with PSANet^30^ is implemented. Through the supplementation of multi-decoder experiments, the algorithm’s accuracy is significantly improved.

## Materials and methods

### Dataset

**Figure 1:**
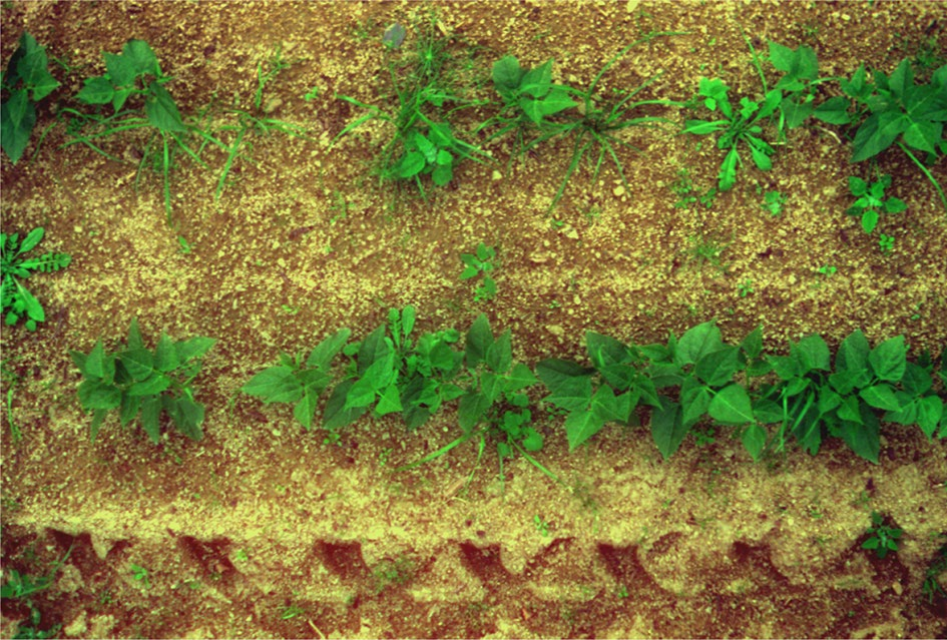
Original image.

**Figure 2:**
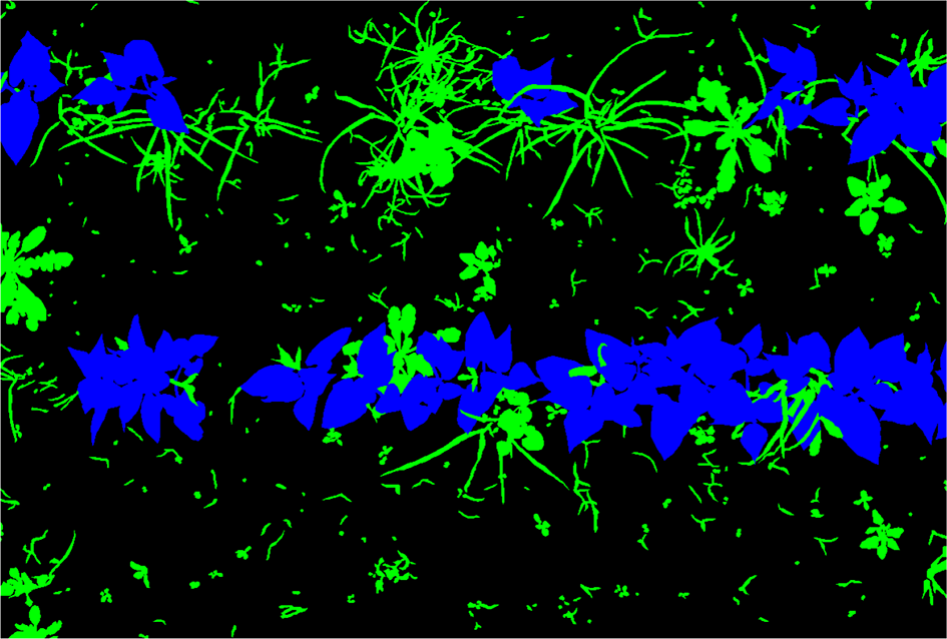
Ground truth.

The dataset used in this paper is sourced from a public repository (10.15454/JMKP9S). This dataset originates from a genuine bean sprout cultivation base in Avignon, France (latitude 46^∘^20’30.3” N, longitude 3^∘^26’33.6” E). The collection equipment utilized is a multispectral camera with six bands at 450/570/675/710/730/850 nm. The images showcase green bean alongside various native weeds such as yarrows, amaranth, geranium, plantago, etc. The collection conditions include rainy days, cloudy days, various lighting conditions, and different time periods. The dataset contains a total of 300 folders, each of which contains one original image and one spectral image collected at 570nm, named as “false.png” and “image.tiff” respectively. There are two label files corresponding to the original images, named as “gt.png” and “gt.xml” respectively. In addition, there is one black-and-white image of green beans and weeds (not available in some folders) named as “index.png”. A total of 1376 files are contained in all the folders. After manually sorting the required “false.png” and “gt.png” for this study, we obtained a total of 300 pairs of bean crop and weed images and their corresponding label images. The images and annotations of the data set are shown in Figs. 1 and 2. The dataset is comprehensive and possesses all the elements required for our experiments. The image quality is high, and the labels are fine-grained, which provides a high degree of reliability for the experiments. In the experiment, the dataset is divided into training, testing, and validation sets at a ratio of 7:2:1, nine offline data augmentation techniques were totally employed (These are horizontal flip, vertical flip, rotate, translate, crop and pad, rotate and crop, gaussian blur, sharpen, and brightness) to address the issue of the dataset being too small. At the same time, data enhancement generates new images that are similar to the original images but have different characteristics. Taken together, these image data enhancements can help the model better understand and adapt to various changes, thereby improving its performance and generalization capabilities.

### The structure of the EPAnet

**Figure 3:**
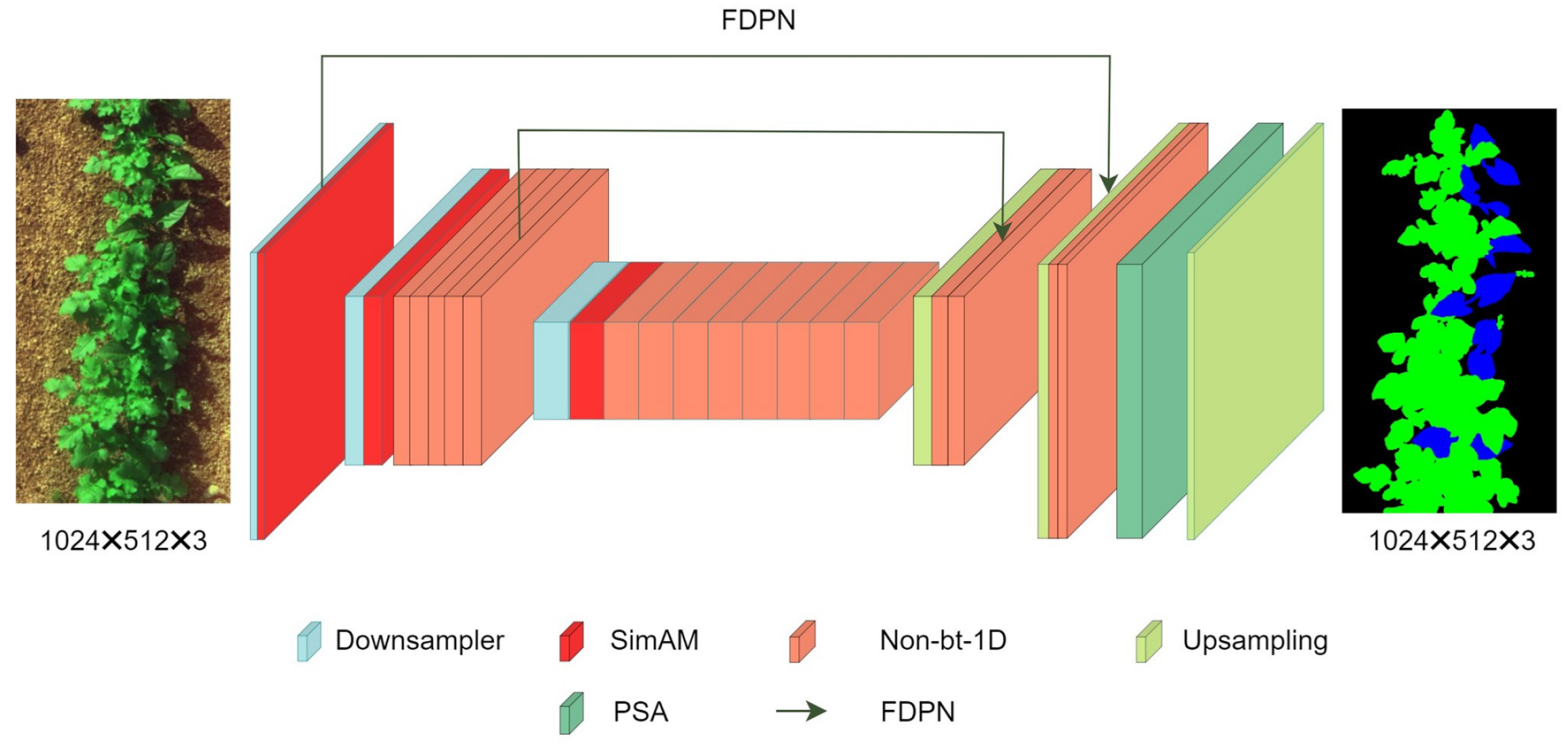
The structure of the EPAnet. Input, output (segmentation category), the feature size obtained at each layer and the input size are not fixed, the corresponding feature mapping is related to the input size.

In this section, we describe the network architecture of EPAnet, which is an improvement based on the ERFnet^31^. To optimize convergence during model training and enhance the overall performance of the model, we adopted a novel loss function that combines the cross-entropy loss function with the Dice loss function. And aiming to achieve the goal of elevating precision, we executed several optimizations on the benchmark model. The overall structure of EPAnet is illustrated in Fig. 3.

EPAnet has 24 layers, with layers 1 to 16 being the Encoder part and layers 17 to 24 as the Decoder. The Encoder is divided into three stages: First stage: The input passes through the Downsampler and SimAM. Second stage: The output from the previous part is further processed by downsample, SimAM, and five Non-bottleneck-1D (Non-bt-1D) layers^31^. Third stage: The output from the second part is further processed by downsample, SimAM, and eight Non-bt-1D layers. The Non-bt-1D balances accuracy and parameter quantity, achieving better context information than Bottleneck. And the experimental Decoder is also divided into three stages: First stage: The output from the Encoder is first processed by upsampling and two Non-bt-1D layers. Second stage: The output from the first stage continues to be processed by upsampling and two Non-bt-1D layers, then passed to the PSA decoder head^30^. Third stage: The data is finally processed by upsampling to produce the output results. In summary, this structure is more balanced, and experimental results indicate a clear improvement in model accuracy.

#### Encoder

After the image is fed into the model, similar to ERFnet. The data goes through a 3 $\times$ 3 convolution with a stride of 2, followed by a max-pooling layer for downsampling, then processed by SimAM, and also utilizing the proposed FDPN connection layer to process the image. These advancements have made a significant contribution to our aim of enhancing segmentation precision.

#### Non-bottleneck-1D

Non-bottleneck-1D is a newly proposed design for a residual layer, aiming to enhance learning capacity and efficiency. It utilizes the advantages of residual connections, adopts sparsity to accelerate and diminishes parameters of Non-bottleneck structures.

#### SimAM^32^

The attention mechanism emulates the concentration exhibited by the human brain on particular information throughout the cognitive process. In complex computational scenarios, selecting valuable information can effectively save computational resources^33^. At present, there are primarily two types of attention mechanisms: spatial attention and channel attention. Spatial attention zeroes in on specific spatial positions and resultant features but may not be particularly attuned to inter-channel communication. Conversely, channel attention might neglect the interplay of spatial information. For optimal outcomes, it’s typically essential to combine both these attention forms^34,35^. The CBAM attention mechanism encompasses both spatial and channel attention and, by adopting max-pooling structures, it has made a significant contribution to model performance improvement. However, integrating these two attentions might increase computational load and could even lead to model convergence issues. Bahdanau, D. et al. proposed the Shuffle Attention module, a method to make the model more lightweight and efficient^36^. Given the enhancement brought about by attention to algorithmic performance, most algorithms now incorporate attention research. After several attempts in this experiment, a suitable method of adding attention was identified.

There is a method to combine BAM (spatial attention module) and CBAM (channel attention module) in parallel or serial manners. In the human brain, these two attention mechanisms often work collaboratively. To more accurately simulate this brain-like attention mechanism, we need to evaluate the importance of each neuron. In neuroscience, information-rich neurons often exhibit different firing patterns compared to their neighboring neurons. When a neuron is activated, it suppresses the nearby neurons, a phenomenon known as spatial inhibitory effect. Neurons with the spatial inhibitory effect appear particularly significant. There are various methods to identify key neurons, one of the most intuitive being measuring the linear separability between neurons. Based on this idea, Yang L. et al. proposed the following energy function:

1 $$\begin{aligned} e_{t} \left( w_{t},b_{t},y,x_{i} \right) = \left( y_{t} -{\hat{t}}\right) ^{2} +\frac{1}{M-1} \sum _{i=1}^{M-1}\left( y_{0} -\hat{x_{i}}\right) ^{2} \end{aligned}$$

The process of minimizing (1) corresponds to enhancing the linear separability between neuron and its peers within the same channel. Utilizing binary labels for streamlining and incorporating a regularization component, the resultant energy function can be articulated as:

2 $$\begin{aligned} e_{t} \left( w_{t},b_{t},y,x_{i} \right) = \frac{1}{M-1} \sum _{i=1}^{M-1} \left( -1-\left( w_{t}x_{i} +b_{t} \right) \right) ^{2}+\left( 1-\left( w_{t} t+b_{t} \right) \right) ^{2} + \lambda w_{t} ^{2} \end{aligned}$$

The ultimately derived analytical solution is as follows:

3 $$\begin{aligned} w_{t}= & {} -\frac{2\left( t-\mu t \right) }{\left( t-\mu t \right) ^{2} -2\sigma _{t} ^{2} +2\lambda } \end{aligned}$$

4 $$\begin{aligned} b_{t}= & {} -\frac{1}{2} \left( t+\mu _{t} \right) w_{t} \end{aligned}$$

Given that all neurons in each channel adhere to the same distribution, one can initially compute the mean and variance on the H and W dimensions of the input features to avoid redundant computations:

5 $$\begin{aligned} e_{t} ^{*} =\frac{4\left( {\hat{\sigma }}^{2} +\lambda \right) }{\left( t-{\hat{\mu }}^{2} \right) +2{\hat{\sigma }}^{2} +2\lambda } \end{aligned}$$

The entire process described above can be represented as:

6 $$\begin{aligned} {\widetilde{X}} =sigmoid\left( \frac{1}{E} \right) \odot X \end{aligned}$$

**Figure 4:**
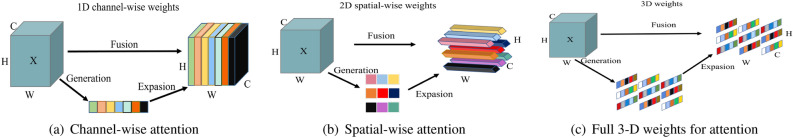
Comparison chart of three different attention generation methods.

SimAM is an efficient attention mechanism, which can enhance network features, improve model performance, and offer enhanced interpretability for the model’s decisions. The integration of SimAM has significantly improved the accuracy of this model. The comparison of the three different attention generation methods is shown in Fig. 4. In Fig. 4a, b, the two approaches can only progressively form attention, but c SimAM can immediately determine three-dimensional weights. In Fig. 4a–c, identical coloring signifies that the same color indicates a unit scalar is used for each channel.

#### FDPN module

The FDPN module is an improvement on the FPN, aiming to address the issue of information loss during the downsampling phase of the original algorithm. In the experiment, the FPN module is incorporated to fill in the lost information, enabling the algorithm to better capture target features at different scales and to achieve information fusion between different levels. In this paper, the proposed FPN module adopts DO-CONV convolution^37^, and this choice significantly enhances the performance of the algorithm. Experimental results show that the model has improved accuracy (detailed experimental results are presented in the ablation study).

#### Decoder

In the decoding layer, we employed a coupled function of cross-entropy loss and Dice loss. Additionally, for structural balance, we appended the PSA Decoder Head after the original Decoder Head. The features captured by the Encoder are processed through deconvolution for upsampling and output.

#### Cross entropy loss

Cross-Entropy loss is a common loss function in multi-class classification tasks. It primarily describes the discrepancy between the actual output probability and the expected output probability. Moreover, the smaller the value of the cross-entropy, the closer the two probability distributions are to each other. Assuming probability distribution as the expected output, as the actual output, and as the cross-entropy, the calculation is as follows:

7 $$\begin{aligned} H\left( p,q \right) =-\sum _{x=1}^{x} \left( p\left( x \right) logq\left( x \right) \right) \end{aligned}$$

#### Dice loss

Dice loss is commonly used in pixel-level semantic segmentation tasks to measure the similarity between predicted results and the actual target. Assuming there are two sets A and B, the calculation formula for the Dice coefficient is as follows:

8 $$\begin{aligned} Dice=\frac{2\cdot \left| A\cap B \right| }{\left| A \right| +\left| B \right| } \end{aligned}$$

Where A represents the model’s predicted results, and B represents the actual target mask (label). Dice loss is derived by converting the Dice coefficient into a loss value through the complement operation.

9 $$\begin{aligned} DiceLoss=1-\frac{2\cdot \left| A\cap B \right| }{\left| A \right| +\left| B \right| } \end{aligned}$$

#### Total loss

The proposed loss function is a combination of cross-entropy loss and dice loss. The cross-entropy loss and dice loss are respectively weighted according to the weight coefficients, and then added together to obtain the final comprehensive loss, as shown in Eq. (10).

10 $$\begin{aligned} TotalLoss=0.6\times \left[ -\sum _{x=1}^{x} \left( p\left( x \right) logq\left( x \right) \right) \right] +0.4\times \left( \frac{2\cdot \left| A\cap B \right| }{\left| A \right| +\left| B \right| } \right) \end{aligned}$$

Coupling the loss functions can integrate the advantages of both, reflecting better generalization capabilities, preventing model overfitting, and also better focusing on the area to be detected. Experimental results demonstrates that our designed loss function performs well (specific results will be detailed in the ablation study).

#### Decoder head

The decoder used by ERFnet is based on the symmetrical structure of Segnet, employing the innovative non-bottleneck-1D derived from convolutional methods and utilizing transposed convolution for upsampling. In this paper, the proposed method incorporates the PSA Head operation following the Decoder Head step.

#### PSA decoder head

**Figure 5:**
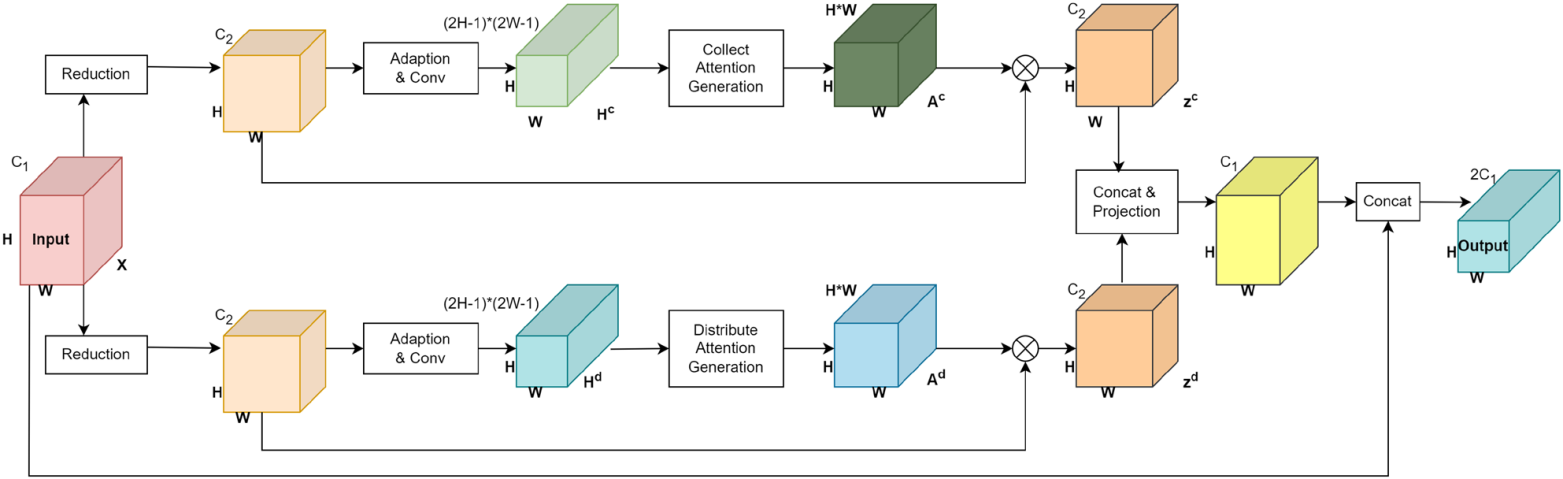
The structural details of the PSA module, the processing flow and output after the data in the previous step enters the PSA.

Convolutional neural networks increase receptive fields by stacking multiple layers, but the effect is not ideal. Some scholars have utilized dilated convolutions to expand receptive fields, thereby enlarging the model’s receptive field. Although dilated convolutions can make the receptive field larger, this operation tends to neglect some information in the image. Additionally, traditional convolution operations confine information flow to local regions, leading to a lack of connection between local and global information. Based on the reasons above, we added PSANet to the second-to-last layer of EPAnet. PSANet is a point-wise spatial attention network that can integrate long-range contextual information. The design of this network allows a point in the feature map to be connected to other points through learnable convolutions, thereby integrating information from nearby and distant points. Additionally, PSANet is designed with bidirectional information flow, enhancing its ability to understand complex scenes. However, PSANet consumes a large amount of memory. Considering the issue of model parameter count, we introduced PSANet in the second-to-last layer of EPAnet to enhance the model’s performance. The Decoder Head of PSANet as shown in Fig. 5, provides a detailed description of the data processing flow of the PSA Decoder Head. In the model, the two branches (upper and lower) are entirely symmetrical, with the upper being the Collect branch and the lower the Distribute branch.

The algorithm input is defined *t* as a three-dimensional data X: H (height), W (width), and $C_{\text {1}}$ (number of channels). (Given that both branches are the same, we will only detail the Collect branch here.) X first goes through a 1$\times$1 convolution to adjust the number of channels, reducing the computational load. The reduced data is then fed into the “Adaption & Conv” module. Using a 1x1 convolution layer, a new feature map $H^{c}$, of size *H*$\times$*W*$\times$*(2H-1)(2W-1)* is obtained.Through the above methods, we can obtain $H^{c}$. The output data from the “Adaption & Conv” module is channeled to the “Collect Attention Generation” module (This module is responsible for generating a new Attention Map, denoted as $A^{c}$, for each position). The conversion process from $H^{c}$ to $A^{c}$ will be explained in detail in the next paragraph. Integrating the above steps, the produced attention weights are combined with corresponding data through a weighted fusion to obtain $Z^{c}$ and $Z^{d}$. Both $Z^{c}$ and $Z^{d}$ enter the “Concat & Projection” module separately. They are then linked with another segment having dimension $C_{\text {1}}$. This process involves integrating data from different origins and performing a linear transformation and projection. And all data streams converge in a module labeled “Concat”. The output is characterized as a block with dimensions H, W,and 2$C_{\text {1}}$.

**Figure 6:**
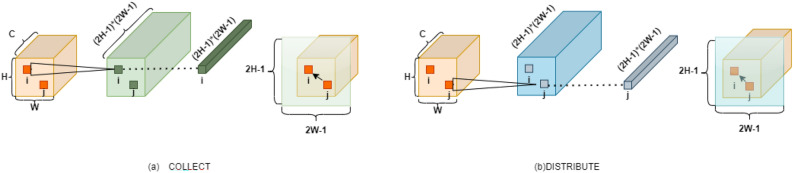
The diagram of point-wise spatial attention, collect attention and distribute attention generation process.

Figure 6 mainly illustrates how the original information in Fig. 5 is converted to $H^{c}$, and the method of converting $H^{c}$ to $A^{c}$. The size of the module $H^{c}$ is *H*$\times$*W*$\times$*(2H − 1)(2W − 1)*, Therefore, each position i in H corresponds to a feature map of size *1*$\times$*1*$\times$*(2H − 1)(2W − 1)*, it can be reshaped into a *(2H − 1)*$\times$*(2W − 1)* feature map, After that, calculate the positional relationship between i and j, but only a part of it is functional in the feature map (as shown by the dashed part in Fig. 6a). The size is *H*$\times$*W*. At the same time, $H^{c}$ can have *H*$\times$*W* positions i. By analogy, *H*$\times$*W* similar feature maps will be generated. The reorganization of the feature maps is $A^{c}$.(Fig. 6 illustrates the spatial attention generation method in the PSA Decoder head).

This module distinctly marks the most evident improvement of the entire experiment. Compared to the baseline model, this module’s Overall Accuracy improved by 0.36%, mIoU by 1.15%, and FWIoU by 0.65%.

### Ethical statement

Plant sampling complies with the IUCN Policy Statement on Research Involving Species at Risk of Extinction and the Convention on the Trade in Endangered Species of Wild Fauna and Flora.

## Experiments

### Experimental setup

In this study, all experiments were conducted in a uniform setting to ensure both objectivity and validity of the results. Throughout the experiments, the operational system employed is Ubuntu 22.04.2 LTS, powered by Intel(R) Xeon(R) Silver 4110 CPU @ 2.10GHz, and equipped with an NVIDIA GTX3090 graphics card and 24GB VRAM. The programming language used is Python 3.8.17, and the deep learning framework is PyTorch 1.10.0 and CUDA 11.1. The experimental learning rate is set to 0.0025, and the number of epochs is set to 400.

### Evaluation metrics

To comprehensively quantify the segmentation performance of different networks, commonly used evaluation metrics in image segmentation were employed, including precision, recall, F1-Score, Overall Accuracy, IoU, mIoU, and FWIoU. It should be noted that:

TP (true positives) denotes the count of pixels which the model accurately identified as the positive category.

FP (false positives) represents the number of pixels that the model misclassified as the positive category, even though they don’t belong to it.

FN (false negative) refers to the number of pixels that should have been predicted as a certain class but were not predicted as that class.

TN (true negative) indicates the number of pixels that the model correctly predicted as belonging to the negative class.

Precision: a higher precision means that the model is more accurate when predicting as positive, reducing the risk of false alarms. However, a higher precision may result in more missed detections. Therefore, in some cases, it’s necessary to consider other evaluation metrics, such as recall, to balance the trade-off between precision and recall. The calculation formula is as follows:

11 $$\begin{aligned} \textrm{Precision} =\frac{TP}{TP+FP} \end{aligned}$$

Recall is another important metric used to measure the performance of a classification model, particularly suitable for binary classification problems. Recall measures the proportion of actual positive samples that the model successfully predicts as positive. The calculation formula for recall is as follows:

12 $$\begin{aligned} \textrm{Recall} =\frac{TP}{TP+FN} \end{aligned}$$

Recall quantifies the extent to which the model covers positive samples, meaning how many actual positives are recognized by the model. A higher recall indicates that the model can capture positives well but might also lead to more false positives. In some applications, such as disease diagnosis, the importance of recall becomes paramount because missing a true positive might have serious consequences. And a balancing act exists between recall and precision. Increasing the model’s recall often decreases precision because capturing more positives might lead to some negatives being incorrectly predicted as positives. Therefore, in practical applications, based on the characteristics of the task, one should consider both recall and precision, choosing an appropriate threshold or other methods to balance these two metrics.

F1-score represents the harmonic average of Precision and Recall. The formula for calculating F1-score is:

13 $$\begin{aligned} \textrm{F1} =2\times \frac{\mathrm {Precision\times Recall} }{\mathrm {Precision\times Recall} } \end{aligned}$$

Where the range of F1-score is [0, 1]. A F1-score close to 1 indicates excellent classifier performance, whereas a score close to 0 indicates poor performance. F1-score offers an integrated measure to assess the balanced performance of a model in both aspects.

Overall accuracy stands as one of the most straightforward and intuitive measures for evaluation. For semantic segmentation, the calculation formula for overall accuracy is:

14 $$\begin{aligned} Overall Accuracy=\frac{TP+TN}{TP+TN+FP+FN} \end{aligned}$$

While overall accuracy is a simple and intuitive metric, it might mask the model’s weaknesses in certain specific categories. If a category’s pixel count is much lower than others, then even with completely wrong predictions for that category, the overall accuracy might remain high. In practice, other evaluation metrics, such as the F1 score or IoU (Intersection over Union), are often considered to achieve a more comprehensive assessment of model performance.

The Intersection over Union (IoU) represents the ratio of the intersection to the union of the model’s predicted results for a specific category and its true values. For object detection, IoU pertains to the ratio between the detected bounding box and the actual box. For image segmentation, it calculates the ratio between the predicted mask and the true mask. The calculation formula is as follows:

15 $$\begin{aligned} IoU=\frac{TP}{TP+FP+FN} \end{aligned}$$

mIoU is the intersection of the predicted area and the actual area divided by the union of the predicted area and the actual area. This calculation gives the IoU for a single category. Repeat this algorithm to calculate the IoU for other categories and then compute their average. It signifies the model’s ratio of intersection to union for each category’s predicted results and true values. The calculation formula is as follows:

16 $$\begin{aligned} mIoU=\frac{1}{N} \sum _{i=1}^{N} IoU_{i} \end{aligned}$$

Where N is the total number of categories. mIoU is a commonly used evaluation metric in image segmentation tasks, reflecting the model’s ability to segment different category targets. A higher mIoU indicates that the model can more accurately segment different object categories. However, one must acknowledge that mIoU might not adeptly discern the category imbalances, meaning certain categories may be rarer than others. Under such circumstances, FWIoU can offer a more accurate representation of each category’s significance.

FWIoU is an enhancement of mIoU, taking into account the influence of category frequency on mIoU. It is especially suitable for addressing imbalanced category distributions. Assuming there are a total of N categories, with the Intersection over Union for each category being IoU1, IoU2, ..., IoUn and the pixel count (frequency) for each category being N1, N2, ..., NN, the calculation formula for FWIoU is:

17 $$\begin{aligned} \textrm{FWIoU} =\frac{\sum _{i=1}^{N} \textrm{IoU} _{i} \times N_{i} }{\sum _{i=1}^{N} N_{i} } \end{aligned}$$

where FWIoU factors are in the product of the Intersection over Union for each category with its frequency in the image. The combined product across categories, divided by the sum of all pixels, results in an mIoU that holistically accounts for frequency. The advantage of FWIoU is that it offers a more equitable assessment for imbalanced category distributions. Categories appearing more frequently will dominate the evaluation outcomes, whereas those less frequent will have a diminished influence. This ensures that the evaluation results better reflect the actual performance of each category. In conclusion, FWIoU is a performance metric for image segmentation models that considers category frequency, effectively addressing imbalanced category distributions.

## Results

### Ablation study

**Table 1:** Result of ablation experiment.

Methods	Classes	Precision (%)	Recall (%)	F1 score (%)	Overall accuracy (%)	iou	mIoU	FWIoU
ERFnet (cross entropy loss)	Background Bean seedling Weed	98.41 87.48 91.38	98.29 87.21 92.09	98.35 87.34 91.73	96.58	96.75 77.54 84.73	86.34	93.59
ERF + PSA (cross entropy loss)	Background Bean seedling Weed	98.55 89.17 92.19	98.59 87.83 92.68	98.57 88.49 92.43	96.94	97.19 79.36 85.93	87.49	94.24
ERF + PSA + double loss	Background Bean seedling Weed	98.75 88.65 92.15	98.49 88.68 93.40	98.62 88.66 92.77	97.04	97.27 79.64 86.51	87.81	94.41
ERF + PSA + double loss + SimAM	Background Bean seedling Weed	98.76 89.36 92.38	98.70 87.83 93.43	98.73 88.59 92.90	97.14	97.49 79.51 86.74	87.92	94.61
ERF + PSA + double loss +SimAM +FDPN	Background Bean seedling Weed	98.89 90.90 91.61	98.70 87.26 94.39	98.79 89.04 92.98	97.23	97.62 80.25 86.88	88.25	94.78

Add different modules one by one to evaluate the impact and role of each module on the model performance. The data in the table are the optimal results of the experiment.

To evaluate the impact of each module on the segmentation performance, we employed an ablation method to test each network module, using ERFnet as the benchmark model and progressively introducing different modules for the ablation study. The outcomes of the ablation experiment are demonstrated in Table 1. First, both our baseline experiments and the experiments with the PSANet module used the Cross Entropy Loss, as shown in our ablation experimental Table 1. ERFnet (cross entropy loss) and ERF+PSA (cross entropy loss) indicate that we used the cross entropy loss as the loss function when conducting ERFnet and ERF+PSA experiments, upon the addition of the PSA module, the metrics of Overall Accuracy, mIoU, and FWIoU improved to 96.94%, 87.49%, and 94.24% respectively. The enhancement is particularly substantial with mIoU witnessing a growth of 1.15%. Second, By incorporating a dual loss function, we can observe that the Overall Accuracy has increased from 96.94 to 97.04%, and the mIoU has increased from 87.49 to 87.81%, compared to the previous experimental results. We then incorporated the SimAM, which improved the mIoU to 87.92% and the Overall Accuracy to 97.14%. Last, after the integration of the FDPN module, compared with the benchmark algorithm, the Overall Accuracy increased by 0.65%, mIoU by 1.91%, and FWIoU by 1.19%. Moreover, each time an innovative aspect was introduced, there was an enhancement in the F1-score. Based on the results in Table 1, after incorporating these network modules, our proposed algorithm evidently outperforms the original benchmark in terms of segmentation on this dataset. This conclusively attests to the successful enhancements we made to the reference model.

### Comparison with different network architectures

To better demonstrate the superiority of our experiment, we further reproduced the current mainstream algorithms, conducted comparative experiments, and presented the comparison results combined with the introduced evaluation metrics. The table below clearly indicates that our proposed method boasts an accuracy rate of 97.23% and an mIoU of 88.25%, excelling in other evaluation criteria as well. Considering that we are conducting a three-class task, we observed that among all categories, the accuracy and recall rate of the bean sprouts are generally lower, indicating that segmenting bean sprouts is a significant challenge. In terms of bean sprout accuracy, our model holds a pronounced edge over others, surpassing them by varying margins between 7% and 11%. In terms of recall rate for bean sprouts, our algorithm performs the best, reaching 87.26%. In contrast, the lowest is the FCN model at just 83.25%, meaning our model has a relative improvement of 4.01%.

**Table 2:** Evaluation results of the proposed EPAnet algorithm compared with current algorithms. Data represents the best experimental results.

Methods	Classes	Precision (%)	Recall (%)	F1 score (%)	Overall accuracy (%)	iou	mIoU	FWIoU
Icnet	Background Bean seedling Weed	98.02 79.45 87.66	97.18 83.73 89.51	97.60 81.53 88.58	95.15	95.30 68.82 79.49	81.20	91.05
UNet	Background Bean seedling Weed	98.52 83.16 88.19	97.83 83.37 91.43	98.17 83.26 89.78	95.89	96.42 71.33 81.46	83.07	92.40
FCN	Background Bean seedling Weed	98 82.14 97.17	97.15 83.25 90.70	97.57 82.69 93.82	95.25	95.26 70.49 80.02	81.92	91.22
Deeplabv3	Background Bean seedling Weed	98.02 83.39 88.65	97.41 85.09 90.71	97.71 84.23 89.67	95.57	95.53 72.76 81.27	83.18	91.78
Fast_scnn^38^	Background Bean seedling Weed	98.41 82.25 89.10	97.67 85.65 90.98	98.04 83.92 90.03	95.87	96.16 72.28 81.87	83.44	92.33
Deeplabv3+	Background Bean seedling Weed	98.47 83.80 90.11	97.87 87.12 91.45	98.17 85.43 90.78	96.19	96.42 74.56 83.10	84.70	92.88
ERFnet	Background Bean seedling Weed	98.41 87.48 91.38	98.29 87.21 92.09	98.35 87.34 91.73	96.58	96.75 77.54 84.73	86.34	93.59
EPAnet (ours)	Background Bean seedling Weed	98.89 90.90 91.61	98.70 87.26 94.39	98.80 89.04 92.98	97.23	97.62 80.25 86.88	88.25	94.78

In terms of weeds, our algorithm achieved a recall rate of 94.39%, which is 4.88% higher than the Icnet model^39^, which has the lowest recall rate. And in terms of Overall Accuracy, the Icnet model performs at 95.15%, while other mainstream algorithms are at a similar level. However, our algorithm achieved 97.23%, a 2.08% increase. As shown in Table 2, in terms of the FWIoU metric, among all mainstream algorithms, Icnet performs the lowest at 91.05%, while our proposed EPAnet model achieves 94.78%, surpassing the Icnet model by 3.73%.

**Figure 7:**
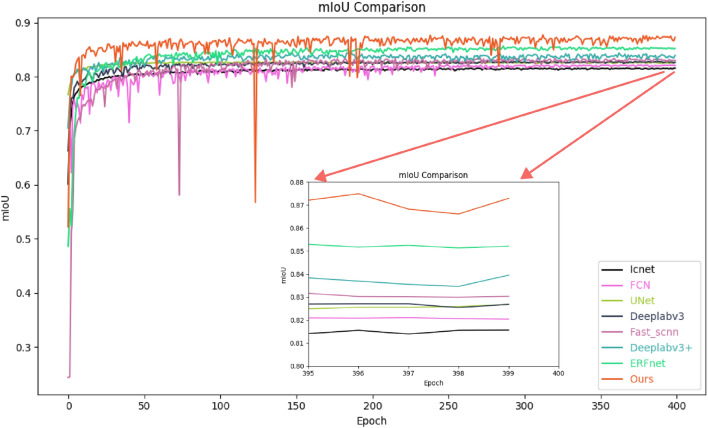
Diagram of mIoU comparison between EPAnet and other models. The enlarged view of the data at the tail of the experiment is shown in the center of the image.The model name and corresponding color lines are displayed on the right.

**Figure 8:**
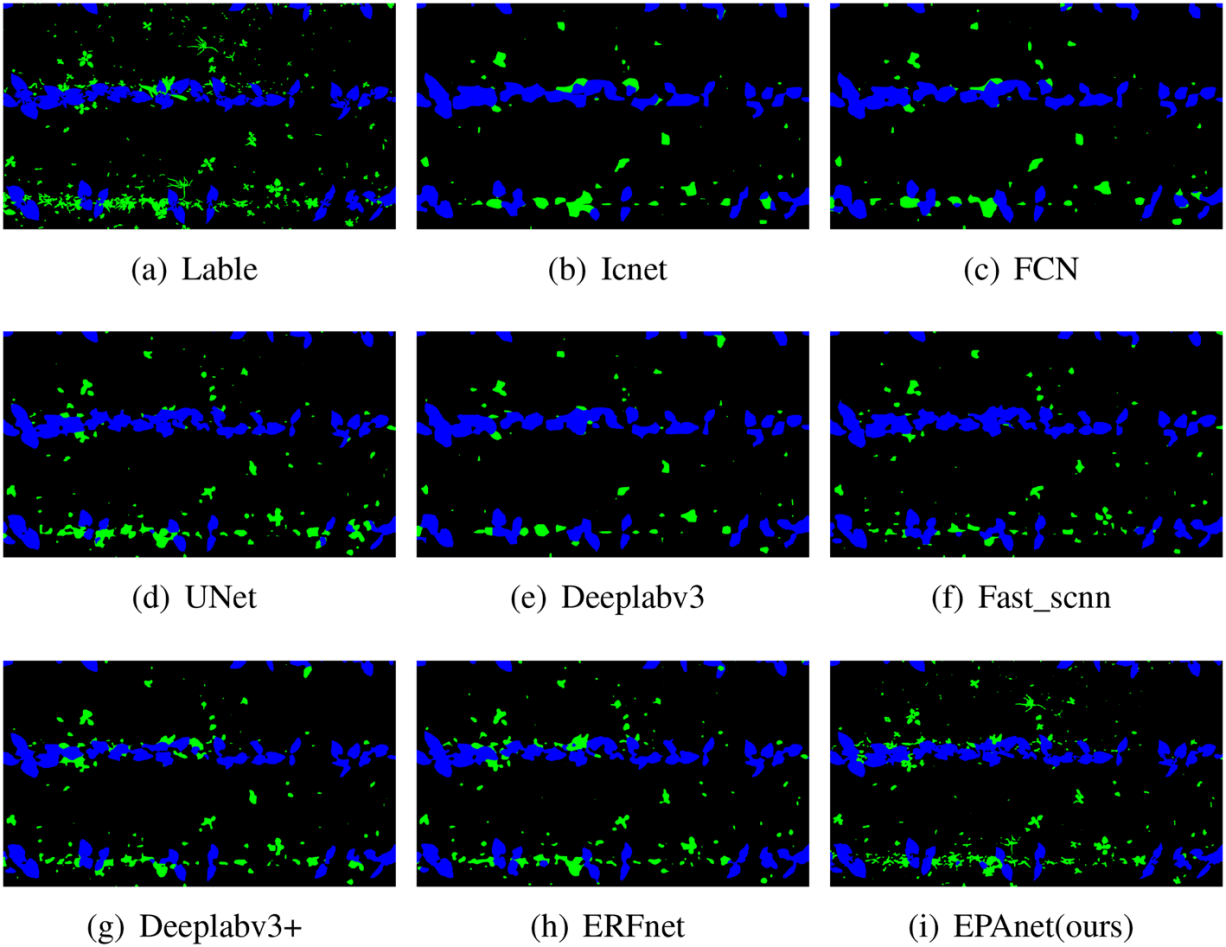
Comparison of segmentation visualization effects of original labels with EPAnet and other contrastive models.

As shown in Fig. 7, to demonstrate the performance comparison between the proposed method and other algorithms, we employ the mIoU line graph during the training process for an intuitive comparison. Due to the involvement of multiple comparative algorithms in this experiment, to better showcase the details, we detailed subfigures in the chart. It is evident that the proposed algorithm significantly outperforms other algorithms in terms of mIoU performance, indicating that our algorithm exhibits superior segmentation results. As shown in Fig. 8, compared to the subpar segmentation results of other models, the proposed EPAnet model excels in tackling segmentation challenges such as leaf edge detection and small object recognition. Its segmentation is very close to the actual Labels, demonstrating the superiority of the EPAnet algorithm.

## Conclusions and discussions

This paper proposes a multi-decoder architecture algorithm based on the ERFnet algorithm, suitable for weed and crop segmentation under natural conditions. Compared to existing segmentation algorithms, it delivers the best results. The method also showcases superior performance under diverse circumstances like different weather patterns, light conditions, and overlapping of leaves. The proposed coupled dual-loss function improves the model’s focus on vital categories, achieving a higher mIoU performance. In comparison to other models, our multi-Decoder Head design captures a broader range of data and recognizes dynamic correlations among them, ensuring more precise segmentation outcomes. And compared to the baseline model, the Overall Accuracy increased by 0.65%, mIoU rose by 1.91%, and FWIoU improved by 1.19%.

Our study demonstrates the effectiveness of our model on mung bean and weed datasets. However, its generalizability to other legume crops and weeds might be limited. Additionally, the required distance between camera and objects for optimal image quality hinders its applicability in drone-based agriculture. Future research will focus on enriching data resources by creating dedicated mung bean and weed datasets to enhance model performance. Furthermore, we aim to optimize the model for edge devices by simultaneously reducing its parameter count and improving evaluation metrics, ultimately leading to faster real-time image segmentation. We will also investigate the optimal weighting scheme for combining cross-entropy and Dice coefficient loss functions, along with exploring the potential integration of reinforcement learning to reduce reliance on labeled data.

## Data Availability

The datasets used and/or analysed during the current study available from the corresponding author on reasonable request.
